# Microbial Volatile Organic Compounds (MVOCs) – rare cause of occupational asthma in two veterinarians

**DOI:** 10.1016/j.rmcr.2026.102377

**Published:** 2026-01-21

**Authors:** Andreas Hoheisel, Ivan Cherrez-Ojeda, Kateryna Gashynova, Jörg Winkler, Gerhard Hoheisel, Daiana Stolz

**Affiliations:** a Clinic of Pneumology, Medical Center-University of Freiburg, Faculty of Medicine, University of Freiburg, Freiburg, Germany; b Institute for Allergology Charité – Universitätsmedizin Berlin, Corporate Member of Freie Universität zu Berlin, Berlin, Germany; c Respiralab Research Group, Guayaaquil, 090507, Ecuador; d Pneumological-Allergological Practices / Study Centers, Leipzig, Germany

**Keywords:** MVOCs, Occupational asthma, Veterinarians

## Abstract

Animal farming is associated with exposure to organic dust containing allergens, endotoxins, and alive microbial organisms. Microbial Volatile Organic Compounds (MVOCs) produced mainly by fungi and bacteria pollute the air and accumulate particularly in confined animal buildings, posing serious health hazards to workers, including veterinarians. The exposure can lead to rhinitis, chronic bronchitis, asthma, and chronic obstructive pulmonary disease (COPD). In a range of cross-sectional studies, wheeze and asthma were associated with exposure to swine, dairy cattle, horse, and sheep, but also to more specific exposures like manure. Occupational asthma has been reported in agricultural workers, including veterinarians. We report two cases of veterinarians who developed asthma in relation to prolonged and intense contact with sheep. No specific allergens could be found, MVOCs were considered as the causative agents.

## 1 Introduction

Animal farming is associated with exposure to organic dust containing allergens, microbial matter including alive microorganisms like fungi, bacteria, and viruses, endotoxins, and factors like irritant gases such as ammonia and disinfectants [1,2]. Many of the components of these bio-aerosols in stables have been identified as pathogen- or microbial-associated molecular patterns (PAMPs/MAMPs), binding to specific receptor molecules and activating innate immunity pathways [1,3]. These bioaerosols, containing a variety of compounds formed during metabolism of fungi and bacteria, have also been termed Microbial volatile organic compounds (MVOCs) [4]. MVOCs can cause a variety of effects including growth inhibition and promotion in interactions between different species, such as fungi. Chemically, the MVOCs identified so far are alcohols, ketones, terpenes, esters, lactones, hydrocarbons, aldehydes, and sulfur and nitrogen compounds. However, the complex cocktail varies temporarily and changes with temperature, substrate and other environmental variables for each microbial species [5]. The air contaminated with MVOCs produced at the breakdown of organic matter of animal origin can have toxic, irritating, and even carcinogenic effects. They are irritants for the mucous membrane of the eyes, nose, throat, bronchial system, and skin [6]. Long-term exposure to MVOCs contributes to compromised immune function in humans and leads to numerous diseases including impairment of lung function [[7], [8], [9], [10]].

Air contaminated with MVOCs pollutants, accumulated particularly in confined animal buildings, can pose serious health hazards to workers, including veterinarians. The exposure has been identified as specific agents/risk factors of asthma, rhinitis, chronic bronchitis, and chronic obstructive pulmonary disease (COPD) [1]. We report two cases of veterinarians who developed asthma in relation to prolonged and intense contact working with sheep.

## 2 Case reports

### 2.1 Case 1

**Fig. 1:**
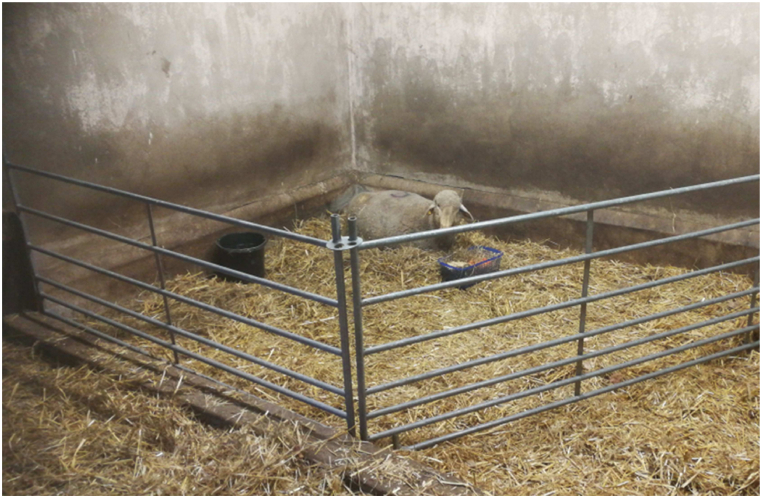
Stable with sheep.

A 38 years old veterinarian, ex-smoker (4 pack years), with bronchial asthma of suspected occupational origin was seen in August 2022 for an expert assessment. Employed in small animal practices 12 years earlier, she noticed work related (WR) rhino-conjunctival allergic symptoms. Specific Immunoglobulin E (sIgE) for cat and dog were positive (Table 1 a). To avoid contact she resumed work teaching animal keepers. Two years later she started working in animal research with sheep and swine, when WR rhinitic symptoms and, for the first time, cough was noticed. The stable where the sheep were kept was old and very moist (Fig. 1). Two of the sheep became gravely ill needing intensive care for up to 6 h daily. The longer she stayed in the stable the more pronounced bronchitic symptoms, cough, shortness of breath, chest tightness, and wheezing became. After work the symptoms subsided. After the sheep died, the stable was closed, post mortem high mold toxins and bacteria were found in bile and ascites of the animals. The occupational health check demonstrated an increased total IgE but no elevation of sIgG levels and WR asthma was suspected (Table 1 b).

**Table 1:** Case 1 – laboratory results.

**a) April 2010 General Practitioner**

- *sIgE (CAP class): positive:* cat epithelium (6), dog scales (2); *negative:* cow, dog, horse epithelium

**b) July 2020 Occupational Physician**

- *Total IgE IU/l:* 210 (<100) - *sIgG (mg/l): normal: Aspergillus fumigatus, Aspergillus niger*

**c) August 2020 Pulmonary Physician**

- *FeNO (ppb):* 7 (<25) - *Eos (% total WCC/absolute number Gpt/l):* 3 (<5.5)/0,2 (0.0–0.5) - *SPT: positive:* mildly HDM II (no animal allergens tested) - *Total IgE U/l:* 188 (<77) - *sIgE (CAP class): positive:* cat (5), dog (3), horse (2); *negative:* mold spores, rat, sheep, HDM I/II

**d) August 2022 Expert Assessment**

- *FeNO (ppb):* 20 (<25) - *Eos (% WCC/absolute number Gpt/l):* 2.4 (<5,5)/0.2 (0,0–0,5) - *SPT: positive:* cat, dog (mildly); *negative:* guinea pig, timothy grass, barley, oat, rye, wheat, mugwort, nettle, dandelion, plantain, alder, hazel, elm, oak, plane tree, ash, ambrosia, HDM I/II, *Alternaria tenuis, Aspergillus fumigatus, Penicillium notatum* - *Total IgE U/ml:* 196 (<87) - *sIgE (CAP class): positive:* cat scales (5), dog scales (3), horse scales (2); *negative:* guinea pig, swine epithelium*,* rat (epithelium, serum), mouse (epithelium, urine), sheep epithelium, budgerigar (extrements, feathers), *Alternaria alternata, Penicillium chrysogenum, Cladosporium herbarum, Aspergillus fumigatus, Candida albicans* - *Total IgG (g/l):* normal - *sIgG (mg/l): normal: Micropolyspora faeni, Thermoactinomyces vulgaris, Stachybotrys atra*

The consulted pulmonary physician performed a spirometry which was normal (Table 2). Five weeks later the patient was asked to go back to the abandoned stable for a field exposure test. After 15 minutes in the stable cough, watery eyes, and running nose developed. The spirometry performed 5.5 hours later was again normal but the nonspecific inhalation challenge (NSIC) was positive (Table 2). To assess bronchial hyperresponsiveness spirometric parameters and whole-body plethysmography were used. A decrease of 20 % of FEV1 and/or an increase of specific airway resistance (sRtot) of at least 100 % from baseline was considered positive [[11], [12], [13], [14], [15], [16], [17], [18], [19]].

**Table 2:** Case 1 – lung function results.

*JUL 15, 2020:* Spirometry^a^	
FEV_1_ (L)	3.40 (114 % pred.)
FEV_1_/VC IN (%)	90.31

AUG 18, 2020: Spirometry^a^ AUG 18, 2020	
FEV_1_	3,54 (119 % pred.)
FEV_1_/VC IN (%)	90.13

*AUG 18, 2020:* NSIC, Bodyplethysmography^a^			
	FEV_1_ (L)	FEV_1_ % pred. (%)	sRtot (kPa x s/L)
Predicted	2.98	–	0.96
Baseline	3.54	118.94	0.47
NaCl	not done	not done	not done
0,2 mg MCh	3.38 (-4.7 %)^b^	113.39 (-4.7 %)^b^	1.72 (+261.3 %)^b^
0,4 mg MCh	3.10 (-12.6 %)^b^	104.01 (-12.6 %)^b^	2.75 (+478.8 %)^b^
2 puffs (200 μg) salbutamol	3.49 (-1.6 %)^b^	117.07 (-1.6 %)^b^	0.69 (+44.9 %)^b^

*AUG 30, 2022:* Spirometry^a^	
FEV_1_ (L)	3.44 (110 % pred.)
FEV_1_/VC IN (%)	83.42

*AUG 30, 2022:* NSIC, Bodyplethysmography^a^			
	FEV_1_ (L)	FEV_1_ % pred. (%)	sRtot (kPa x s/L)
Pred.	3.12	–	0,96
Baseline	3.44	110,26	0,91
NaCl	3.42 (-0 %)^b^	109,62 (-0 %)	1,26 (+12 %)
0,1 mg MCh	2.70 (-22 %)^b^	86,54 (-22 %)^b^	2,03 (+82 %)^b^
0,3 mg MCh	1.78 (-48 %)^b^	57,05 (-48 %)^b^	3,26 (+192 %)^b^
2 puffs (200 μg) salbutamol	3.24 (-6 %)^b^	103,85 (-6 %)^b^	1,02 (-9 %)^b^

a By JAEGER Masterscreen Body™.

b Change to baseline; FEV1: forced expiratory in 1 second; MCh: methacholine; Na Cl: normal saline; NSIC: non specific inhalation challenge; sRtot: total specific airway resistance; VC IN: inspiratory vital capacity.

The skin prick test (SPT) was mildly positive for house dust mite (HDM) II only, sIgE for cat, dog, and horse were positive, all other tested allergens including fungi, sheep, and swine were negative (Table 1 c). The fraction of exhaled nitric oxide (FeNO) and eosinophils were normal. The diagnosis of asthma was established and regular treatment with a combination of low dose inhaled corticosteroids (ICS) and long (LABA) and short acting beta agonists (SABA) as needed was initiated.

During the expert assessment visit two years later the SPT was positive for cat and dog, total IgE was increased, sIgE was positive for cat, dog, and horse, all others were negative, including sheep, swine, and fungi, as was total IgG and sIgG for fungi (Table 1 d). The NSIC again was strongly positive (Table 2). FeNO and eosinophils were normal. The diagnosis of asthma and type I allergy against cat, dog, and horse were confirmed. Type III allergy could be ruled out for the allergens tested. MVOCs were considered as the triggering factors of asthma.

Although an allergy to small animals had caused rhino-conjunctival symptoms in the past, WR asthma was only observed following the intense and prolonged exposure to MVOCs. The occupational cause of asthma in this veterinarian was acknowledged by the respective Occupational Authority. She resumed work in animal research with rats, mice, and monkeys wearing protecting gear without any symptoms of allergy or asthma. She avoids mold-contaminated stables and remains well controlled under regular anti-asthmatic treatment.

### 2.2 Case 2

A 42 years old veterinarian, current smoker (9 pack years), colleague of case 1, with bronchial asthma of suspected occupational origin was seen in March 2023 for an expert assessment. She reported mild neurodermitic symptoms since childhood but no history of allergy. Mother and son suffered from asthma, another son from neurodermitis. She noticed WR mild cough starting three to 4 h after exposure to pigs in animal research. Since working with sheep, cough and wheezing developed in the stable, especially when dust was produced by the movement of the animals, in the evening sometimes incessant coughing occurred. The symptoms started mainly thirty to 40 min after exposure and became worst after 4 h. Later additional mild gastric pain, loss of appetite, diarrhea, and mild itching and sometimes erythema of the forearms after close contact with the sheep were noticed. During the weekends and on holiday symptoms subsided. After the sheep fell severely ill, the time spent in the stable became very long and symptoms intensified. Finally, the sheep died. The occupational health check demonstrated an increased total IgE and a mildly elevated mold IgG level (Table 3 a).

**Table 3:** Case 2 – laboratory results.

**a) July 2020 Occupational Physician**

- *sIgE (CAP class): normal: Aspergillus fumigatus, Aspergillus niger, Aspergillus spp. mixture* - *sIgG (mg/l): normal: Aspergillus fumigatus, Aspergillus versicolor; elevated:* slightly *Aspergillus niger* 26 mg/ml (<24)

**b) August 2020 Pulmonary Physician**

- *FeNO (ppb):* 14; one week later (field test) 15 (<25) - *Eos % total WCC/absolute number Gpt/l):* 0,01 (<5.5)/0,1 (0,0–0,5) - *SPT: positive:* timothy grass, rye, mugwort, ambrosia, plantain; *negative:* cat, dog, hamster, rabbit, guinea pig, horse, alder, hazel, birch, ash*,* HDM I/II, *Alternaria tenuis, Cladosporum herbarum, Aspergillus fumigatus* - *Total IgE U/l:* not determined - *sIgE (CAP class): positive:* 1: HDM II; *negative:* cow epithelium, sheep epithelium, rat (epithelium, serum, urine), mouse (epithlium, serum, urine), horse, cat, dog, HDM II, *Alternaria alternata, Aspergillus fumigatus, Aspergillus niger, Fusorium proliferatum, Cladosporium herbarum, Penicillium chrysogenum, Fusarium proliferatum* - *Total IgG: normal* - *sIgG (mg/l): normal: Alternaria alternata*

**c) March 2023 Expert Assessment**

- *FeNO (ppb):* 16 (<25) - *Eos (% total WCC/absolute number Gpt/l):* 2,6 (<5.5)/0,3 (0.0–0.5) - *SPT: positive:* timothy grass, barley, oat, wheat, mugwort, nettle, dandelion, plantain, alder, hazel, elm, oak, plane tree, ash, ambrosia; *negative:* HSM I/II, dog, cat, poplar, willow, birch, beech, *Aspergillus fumigatus, Penicillium notatum* - *Total IgE (U/ml):* not determined - *sIgE (CAP class):* 2: timothy grass, barley, wheat, birch, hazel, oak, elm, ambrosia, mugwort, plantain, nettle; *normal:* dog, cat, sheep epithelium, swine epithelium, rat (epithelium, urine), *Penicillium chrysogenum, Cladosporium herbarum, Aspergillus fumigatus, Alternaria alternata, Aspergillus niger* - *Total IgG (g/l):* normal - *sIgG (mg/l): Elevated:* slightly *Cladosporium herbarum* 37,1 (<37,0); *normal: Alternaria alternata, Aspergillus fumigatus, Aspergillus niger, Penicillium chrysogenum*

The consulted pulmonary physician found the spirometry and NSIC negative (Table 4). FeNo and eosinophils were normal (Table 3 b). A week later after exposure to the old stable in a field test, cough, shortness of breath, and wheezing occurred 20 minutes upon entering. Four hours later the spirometry was normal, however, the NSIC this time was positive (Table 4). SPT showed positivity for grain and herbs, total IgE was increased, sIgEs were negative including sheep and mold (Table 3 b).

**Table 4:** Case 2 – lung function results.

*AUG 18, 2020:* Spirometry^a^	
FEV_1_ (L)	3.42 (104 % pred.)
FEV_1_/VC IN (%)	88.61

*AUG 18, 2020:* NSIC, Bodyplethysmography^a^			
	FEV_1_ (L)	FEV_1_ % pred. (%)	sRtot (kPa x s/L)
Predicted	3.30	–	0.96
Baseline	3.41	103.71	1.07
NaCl	not done	not done	not done
0.2 mg MCh	3.23 (-5,6 %)^b^	97.94 (-5.6)^b^	1.29 (+20.5)^b^
0.4 mg MCh	3.24 (+5,4)^b^	98.13 (-5.4)^b^	1.45 (+35.2)^b^
2 puffs (200 μg) salbutamol	3.32 (-3,0)^b^	100.64 (-3.0)^b^	1.59 (+48.4)^b^

*AUG 25, 2020:* Spirometry^a^	
FEV_1_ (L)	3.38 (102 % pred.)
FEV_1_/VC IN (%)	88.42

*AUG 25, 2020:* NSIC, Bodyplethysmography^a^			
	FEV_1_ (L)	FEV_1_ % pred. (%)	sRtot (kPa x s/L)
Pred.	3.30	–	0.96
Baseline	3.38	102.41	0.76
NaCl	not done	not done	not done
0.2 mg MCh	3.27 (-3.2)^b^	99.11 (-3.2)^b^	0.80 (+5.6)^b^
0.4 mg MCh	2.89 (-14.3)^b^	87.73 (-14.3)^b^	1.10 (+44.4)^b^
0.65 mg MCh	2.98 (-11.8)^b^	90.37 (-11.8)^b^	1.53 (+100.7)^b^
2 puffs (200 μg) salbutamol	3.33 (-1.4)^b^	101.0 (−1.4)^b^	0.57 (−24.7)^b^

MAR 14, *2023:* Spirometry^a^	
FEV_1_ (L)	3.18 (92 % pred.)
FEV_1_/VC IN (%)	82.85

MAR 14, *2023:* NSIC, Bodyplethysmography^+^			
	FEV_1_ (L)	FEV_1_ % pred. (%)	sRtot (kPa x s/L)
Pred.	3.45	–	0.96
Baseline	3.18	92.17	0.85
NaCl	3.17 (-0 %)^b^	91.88 (-1 %)^b^	1.04 (+22 %)^b^
0,1 mg MCh	3.15 (-0 %)^b^	91.30 (-1 %)^b^	1.30 (+53 %)^b^
0,3 mg MCh	2.88 (-9 %)^b^	83.48 (-9 %)^b^	2.34 (+175 %)^b^
2 puffs (200 μg) salbutamol	3.30 (+4 %)^b^	95.65 (+4 %)^b^	0.65 (-24 %)^b^

a By JAEGER Masterscreen Body™.

b Change to baseline; FEV1: forced expiratory in 1 second; MCh: methacholine; Na Cl: normal saline; NSIC: non specific inhalation challenge; sRtot: total specific airway resistance; VC IN: inspiratory vital capacity.

After the stable had been closed (details see case 1) she resumed research again with pigs, which were kept in modern stables, where, compared to the previous situation with sheep, only mild WR symptoms after about 4 h occurred.

During the expert assessment two years and eight months later, the SPT demonstrated positivity for spring pollen allergens, grass, grain, and herbs, sIgEs were positive for the same allergens, however, negative for mold, sheep, and swine. Total and sIgGs were normal (Table 2 c). The spirometry was normal, but the NSIC was positive (Table 4). The diagnosis of asthma was confirmed.

Although type I allergens against spring pollen, grass, and herbs could be found, the patient had never experienced any seasonal rhino-conjunctival or asthmatic symptoms. Together with her mild neurodermitis and positive family history of asthma and neurodermitis she was classified as an individual with atopic disposition, expressing immunological responses to various allergens (increased total and sIgE) but without any rhino-conjunctival or respiratory symptoms. WR asthmatic symptoms only developed with swine and more pronounced with sheep. Neither swine, sheep, nor mold allergens type I, nor type III could be demonstrated. The slightly increased *Cladosporium herbarum* IgG level was considered only demonstrating past contact but not as proof of an allergic type III reaction. The underlying mechanism of the manifestation of asthma in this case was the atopic disposition and the intense and prolonged exposure to MVOCs. The occupational cause of asthma in this veterinarian was acknowledged by the respective Occupational Authority.

The patient is now working as a public veterinarian supervisor inspecting stables and animal breeding facilities. When entering large facilities for chicken, swine, sheep, cows, or sometimes horse stables, she notices cough and shortness of breath roughly 20 min after entering the building. Quite often severe shortness of breath develops immediately upon entering the breeding facility forcing her to retreat at once.

She considers her asthma controlled by inhalation of SABA 1–2 puffs as needed, however, was strongly recommended to take regular ICS daily in order to prevent more severe asthmatic reactions.

The microbiological workup of the old stable showed high degrees of bacteria and mold, especially in hay, feeding trough, drain, stable walls, and floor (Table 5). The massive degree of bacteria and high degree of mold in the feeding trough demonstrates the high degree of bacterial and fungal contamination of the feed for the sheep. All bacteria and mold species found in the stable contributed to the toxic effect of the air polluted by MOVCs.

**Table 5:** Stable – bacteriological and mycological cultures and mass spectrometry results, April 2020.

*Stable walls:* moderate degree of *Pantoea agglomerans,* mild degrees of *Staphylococcus sciuri, Staphylococcus equorum;* no yeast or mold
*Stable floor:* high degrees of *Staphylococcus equorum, Aerococcus viridans,* moderate degree of *Acinetobacter indicus,* no *Clostridium spp.;* no yeast or mold
*Drain:* high degrees of *Escherichia coli, Mycoides odoratimimus, Enterococcus casseliflavus,* moderate degree of *Aerococcus viridans* and *Acinetobacter Iwoffii,* no *Clostridium spp.;* no yeast or mold
*Hay:* high degree of *Bacillus spp.*
*Feed storage walls:* high degree of *Penicillium spp*., low degree of *Bacillus horneckiae*
*Feed storage floor:* high degrees of *Bacillus jeotgalli* and other *Bacillus spp.,* low degree of *Pantoea agglomerans, Acinetobacter spp;* no yeast or mold
*Feeding trough:* massive degrees of *Bacillus mycoides, Bacillus weihenstephaniensis, Bacillus pumillus, Solibacillus Silvestri;* high degree of mold

In summary, both veterinarians developed WR asthma. Case 1 had a type I allergy with mild rhino-conjunctival symptoms in the past when exposed to certain animals. Case 2 had an atopy with mild neurodermitis and increased total and sIgE but with no respiratory symptoms in the past. Both veterinarians developed initially WR mild symptoms of asthma (cough) when exposed to MVOCs, typically only after some time of exposure. Symptoms became much worse in the unusual setting of a stable highly contaminated with mold, bacteria, and high concentrations of MVOCs. The intense and long daily duration of exposure caused in both cases a clinically significant bronchial asthma necessitating long term anti-asthmatic treatment. An allergy to sheep, swine, or mold could be ruled out in both cases. There was also no finding suggestive of a type III allergy leading to hypersensitivity pneumonitis (HP)/exogenous allergic alveolitis (EAA). Both veterinarians are able to continue with their occupation, however, being on long term anti-asthmatic medication and having to avoid contact with MVOCs at their working places.

## 3 Discussion

Mold, a collective term for filamentous cell- (hyphae-) and spore-forming microfungi, are ubiquitous components of our biosphere, found to varying degrees in outdoor air, indoor spaces, and in many workplaces, including agriculture [20]. Indoor air in animal breeding buildings is contaminated with the products of decaying organic matter and those emerging during metabolic processes in the animal gastrointestinal tract [7,[21], [22], [23]]. Common components of the breeding environment are microbial pollutants, such as bacteria, fungi, viruses, and their metabolites. Mold and their toxins are a recognized occupational risk for respiratory diseases [24].

MVOCs are produced by mold and bacteria during the fermentation of fresh or stored animal manure and decomposing spoiled feed [7]. The air contaminated with MVOCs pollutants, especially when accumulated in confined animal buildings, can pose serious health hazards to workers [7,23,25]. Swine farmers, for example, are exposed to mold inside swine housing units associated with heavy exposure to inhalable spores of fungi from the genus *Aspergillus, Penicillum, Fusarium, Stachybotris,* or *Trichoderma,* which are known to cause adverse human health effects. The environment of an animal facility favors rapid microbial multiplication and development, especially when appropriate sanitary and hygiene conditions are not maintained [26]. When inhaled, the bioaerosol with organic compounds derived from microorganisms (endotoxins, peptidoglycans, glucans and mycotoxins) induces an immunotoxic reaction, an allergy-like effect, in the pulmonary immune system [7,27]. Inhaled PAMPs from bio-aerosols have been shown to induce airway inflammation in healthy and asthmatic subjects and symptom exacerbations to a variable degree, likely depending on the burden of exposure and polymorphisms in endotoxin cell receptors and signal transduction molecules [1,3]. In agriculture airborne mycotoxins can cause considerable health effects [26,[28], [29], [30]]. These substances are irritants for the mucous membranes causing symptoms, such as irritation of the eyes, nose and lips, lip ulceration, headache, diarrhea, hoarseness, cough, chest tightness, palpitation, shortness of breath, wheezing, and dry cough [20,[31], [32], [33]]. Symptom intensity is dependent on air pollution concentration [34]. Repeated irritation by MVOCs can induce respiratory diseases like asthma, especially with high concentrations in the air [31,34].

A limitation of our study is the fact that no quantitative exposure assessment of MVOCs levels was performed, and no specification of plausibly implicated compounds could be determined in our cases. However, despite the absence of direct measurement results, the confirmation of significant microbial contamination by environmental sampling, the likely presence of components, e. g. endotoxins, β-glucans, and ammonia - known triggers of irritant-induced asthma -, the temporal symptom pattern, the stable characteristics, and the negative allergen workup still point to MVOCs as the leading suspected cause of asthma in our patients.

The diagnosis of asthma caused by intense and prolonged exposure to MVOCs in both of our cases was based on the symptoms and the positive NSIC in the exposure (field) test four months after the last exposure. It is well known that the sequelae and symptoms of affected individuals can persist even after exposure avoidance [20,35,36].

A typical sign of endotoxin induced inflammation and non specific bronchial hyperresponsiveness (BHR) is the subacute development of symptoms with a delay of 4–8 hours after exposure [1]. Such a delayed onset of symptoms of up to 4 h was also reported by our patients.

Gastrointestinal symptoms as reported by case 2 can be explained by ingestion of mycotoxins, bacterial particles, and endotoxins, and have been reported before [7,21,22,31].

Itching of the skin and erythema developed in case 2 when WR exposure to the animals became intense, likely having been facilitated by the mild neurodermitis in this patient. MVOCs are irritative to mucous membranes and in sensitive patients to skin alike [7,31]. Moreover, her state of atopy, which is a known risk factor for asthma, is likely to have increased her susceptibility to irritant exposures and have facilitated the development of asthma in this patient [37,38].

The underlying mechanism responsible for symptoms like headache, eye irritation, nausea, or skin irritation is not known in many cases. It is suspected that no single component in the airborne emissions can induce such effects but additivity or synergy of combined components may be the cause of these symptoms [31].

New onset asthma in farmers working with pigs and dairy cattle was reported in a Danish study, which found that during the first years in farming school the risk was significantly increased [39]. In a range of other cross-sectional studies, wheeze and asthma were associated with exposure to pigs, dairy cattle, horse, and sheep, but also with more specific exposures like manure [1,40,41]. Most intensively studied are the pathogenic mechanisms of wheezing and asthma in swine farming, especially in confinement buildings, where high and chronic airborne exposures may not only lead to local airway and lung inflammation, but also to systemic effects as shown by increased levels of circulating serum cytokines [1,23,42]. Symptoms are wheeze, cough, and other typical asthmatic symptoms and features like increased BHR in the NSIC [1,[43], [44], [45], [46]].

All these symptoms were present in our patients. Case 1 had years before only mild rhino-conjunctival symptoms when in contact with specific small animals. Only years later mild WR asthmatic symptoms (cough) when doing research with swine developed. Only after intense and long duration of WR exposure to the irritant and toxic effects of MVOCs caring intensely for sheep, significant symptoms developed, necessitating regular anti-asthmatic inhalative treatment. Case 2 can be considered as a new onset asthma. The clinical presentation in this case was facilitated by atopy and developed, as in case 1, initiated by WR intense and prolonged exposure to the irritant and toxic effects of MVOCs. In both cases long term anti-asthmatic treatment is indicated.

Our patients did not show type I or type III sensitizations to sheep, swine, or mold spp. And although negative SPT or sIgE results do not totally exclude sensitizations, the cause was obviously the WR long and intense contact to MVOCs causing symptoms and signs of asthma. The potential of inducing airway inflammation in healthy and asthmatic subjects and symptom exacerbations have been described before [1,3].

Allergic bronchopulmonary aspergillosis (ABPA) must have been considered as a possible differential diagnosis in our patients, considering the symptoms of asthma and the dark and damp sheep pens conducive to the growth of fungi like *Aspergillus spp.,* especially *Aspergillus fumigatus*. However, the diagnostic criteria as laid out in detail in the ABPA clinical practice guidelines of the ERS were not met [47].

Although a link between MVOCs and respiratory health symptoms in humans has been demonstrated by numerous studies, standardized test systems for evaluating the toxicity of MVOCs are currently not available [1,5]. A thorough anamnesis and identification of WR exposure associated symptoms is considered as the key point in the diagnosis of asthma in these cases [1].

Another factor that rendered the two veterinarians to be comparable to workers doing intense farming is the fact that they were for many hours in close contact with the gravely ill animals in a relatively small stable. Studies have shown that personal exposures to toxic and irritant airborne substances are highest during specific stable activities involving feed handling, distribution of bedding, and intense handling of animals [1,[48], [49], [50], [51]] but are lowest during field work [1,52].

Another factor that contributed significantly to the development of WR symptoms in our patients were the poor conditions of hygiene and ventilation in the stable. Studies have shown that feeding, flooring, and ventilation parameters are strong predictors of indoor personal exposure levels to bio-aerosols [1,48,49,[53], [54], [55]].

Poor hygiene and ventilation conditions together with high humidity and moisture in materials and on surfaces in stables contribute to the growth of mold and bacteria causing ample production of MVOCs [20,32,56]. Mold species associated with mold infestation primarily include *Acremonium spp., Aspergillus penicillioides, Aspergillus restrictus, Aspergillus versicolor, Chaetomium spp., Phialophora spp., Penicillium chrysogenum, Penicillium brevicompactum, Scopulariopsis brevicaulis, Scopulariopsis fusca, Scopulariopsis brumtii, Scopulariopsis chartarum, Stachybotrys chartarum, Tritirachium (Engyodontium) album,* and *Trichoderma spp.,* and in rare cases *Aspergillus fumigatus* [20,57].

The contamination of the interior of the stable with bacteria and mold was well documented by cultures and mass spectrometric analyses. The stable walls, floor, drain, and hay showed high degrees of bacterial contamination but no yeast or mold. The feed storage place was contaminated by mold and bacteria, the feeding trough showed massive bacterial and mold contamination explaining why toxins were found in the bile and ascites of the animals postmortem.

The two cases presented show that veterinarians, when in close and prolonged contact with animals, comparable to particular situations of farming, can develop WR symptoms leading to occupational asthma. No allergy of type I or type III of the allergens to which they were exposed, like sheep dander or mold, could be demonstrated. MVOCs, present in high concentrations at the working place, should always be considered as a possible cause of asthmatic symptoms. Although case 1 had a type I allergy with mild rhino-conjunctival symptoms when exposed to certain animals, this mechanism was not primarily involved in the development of asthma. Case 2 had an atopy with mild neurodermitis and symptomless increased total and specific IgE, which might have contributed to the expression of asthma, albeit was not the leading cause. MVOCs present in high concentrations at the working place over a long period of time and in high concentrations potentially leads to the development of asthma. Both veterinarians can resume their profession, with regular medication and by avoiding exposure to MVOCs, especially in confined animal buildings. In summary, a thorough anamnesis and identification of symptoms as clearly exposure-associated is the key point in the diagnosis of WR respiratory diseases in agricultural workers including veterinarians. MVOCs-related asthma should be considered, when asthmatic symptoms develop after having been in close and prolonged contact with animals, especially in confined animal buildings with poor ventilation. Negative allergen testing, type I and III alike, should trigger additional environmental testing.

## CRediT authorship contribution statement

**Andreas Hoheisel:** Writing – original draft, Validation, Resources, Project administration, Methodology, Investigation, Data curation, Conceptualization. **Ivan Cherrez-Ojeda:** Writing – review & editing. **Kateryna Gashynova:** Writing – review & editing. **Jörg Winkler:** Writing – review & editing, Data curation. **Gerhard Hoheisel:** Writing – original draft, Resources, Project administration, Methodology, Investigation, Data curation, Conceptualization. **Daiana Stolz:** Writing – review & editing.

## Statement of ethics

This retrospective review of patient data did not require ethical approval in accordance with local and national guidelines. The patients provided written informed consent for publication of the details of their medical cases and the accompanying image.

## Data availability statement

All data generated or analysed during this study are included in this article. Further inquiries can be directed to the corresponding author.

## Funding sources

No funding was received for this work.

## Declaration of competing interest

The authors declare the following financial interests/personal relationships which may be considered as potential competing interests: None Dr. med. Andreas Hoheisel reports was provided by University Medical Center Freiburg. If there are other authors, they declare that they have no known competing financial interests or personal relationships that could have appeared to influence the work reported in this paper.

## Abbreviations

BHR: Bronchial hyperresponsiveness
CAP: Carrier polymer
EAA: Exogenous allergic alveolitis
Eos: Peripheral blood eosinophils
FeNO: Fraction of exhaled nitric oxide
FEV1: Forced expiratory in 1 second
HDM: House dust mite
HP: Hypersensitivity pneumonitis
ICS: Inhaled corticosteroids
LABA: Long acting beta agonists
MAMP: Microbial-associated molecular pattern
MCh: Methacholine
MVOCs: Microbial Volatile Organic Compounds
Na Cl: Normal saline
NSIC: Non specific inhalation challenge
PAMP: Pathogen-associated molecular pattern
ppb: Parts per billion
SABA: Short acting beta agonists
sIgE: Specific immunoglobulin E
sIgG: Specific immunoglobulin G
SPT: Skin prick test
sRtot: Total specific airway resistance
VC: IN Inspiratory vital capacity
WCC: Peripheral blood white cell count
WR: Work related
